# Peripheral ischemic reserve in sepsis and septic shock as a new bedside prognostic enrichment tool: A Brazilian cohort study

**DOI:** 10.1371/journal.pone.0288249

**Published:** 2023-07-05

**Authors:** Ana Carolina de Miranda, Fernanda do Carmo De Stefani, Bruna Cassia Dal Vesco, Hipólito Carraro Junior, Luis Gustavo Morello, Jamil Assreuy, Igor Alexandre Cortês de Menezes

**Affiliations:** 1 Department of Internal Medicine, Hospital de Clínicas, Federal University of Paraná, Curitiba, Paraná, Brazil; 2 Intensive Care Unit, Hospital de Clínicas, Federal University of Paraná, Curitiba, Paraná, Brazil; 3 Oswaldo Cruz Foundation, FIOCRUZ, Curitiba, Brazil; 4 Department of Pharmacology, Federal University of Santa Catarina, Florianópolis, Santa Catarina, Brazil; Faculty of Medicine Chiang Mai University, THAILAND

## Abstract

Microvascular dysfunctions are associated with poor prognosis in sepsis. However, the potential role of clinical assessment of peripheral ischemic microvascular reserve (PIMR), a parameter that characterizes the variation of peripheral perfusion index (PPI) after brief ischemia of the upper arm, as a tool to detect sepsis-induced microvascular dysfunction and for prognostic enrichment has not been established. To address this gap, this study investigated the association of high PIMR with mortality over time in patients with sepsis and its subgroups (with and without shock) and peripheral perfusion (capillary-refill time). This observational cohort study enrolled consecutive septic patients in four Intensive-care units. After fluid resuscitation, PIMR was evaluated using the oximetry-derived PPI and post-occlusive reactive hyperemia for two consecutive days in septic patients. Two hundred and twenty-six patients were included—117 (52%) in the low PIMR group and 109 (48%) in the high PIMR group. The study revealed differences in mortality between groups on the first day, which was higher in the high PIMR group (RR 1.25; 95% CI 1.00–1.55; p = 0.04) and maintained its prognostic significance after multivariate adjustment. Subsequently, this analysis was made for sepsis subgroups and showed significant differences in mortality only for the septic-shock subgroup, with was higher in the high PIMR group (RR 2.14; 95% CI 1.49–3.08; p = 0.01). The temporal ΔPPI peak values (%) analyses did not demonstrate maintenance of the predictive value over the first 48 h in either group (p > 0.05). A moderate positive correlation (r = 0.41) between ΔPPI peak (%) and capillary-refill time (s) was found within the first 24 hours of diagnosis (p < 0.001). In conclusion, detecting a high PIMR within 24 h appears to be a prognostic marker for mortality in sepsis. Furthermore, its potential as a prognostic enrichment tool seems to occur mainly in septic shock.

## Introduction

Sepsis is a dysregulated host response to infection that causes life-threatening organ dysfunction and remains a significant health challenge. Due to its high morbidity and mortality, the problematic care of septic patients generates undeniable impacts on individuals and health systems, especially in low-and middle-income countries [1]. In these countries, mortality rates between 30 and 80% are often explained by insufficient knowledge of the disease, lack of preventive measures for infections, high prevalence of nosocomial infections, and inadequate intensive care units (ICUs) access and structure [1].

Despite a growing understanding of the pathophysiology of sepsis, several studies have failed to validate effective therapies, even in high-income countries [1]. This scenario points to the need for more personalized management to address the heterogeneity of patients and improve survival [2]. Thus, “prognostic enrichment” becomes a key concept: it refers to selecting patients most likely to have a disease-related outcome of interest [2]. In personalized medicine, such a concept would help the clinician consider the benefit-risk ratio, whereby only therapies carrying minimal risks may be justified for selected patients with a higher likelihood of an outcome [2].

Persistent microcirculatory disturbances commonly occur in sepsis even after the normalization of macro-hemodynamics [3,4]. A recent review confirmed the association with mortality even when evaluated in different tissues such as skin, sublingual, and muscle [5]. Among the mechanisms involved, great emphasis has recently been given to vascular hyporesponsiveness and endothelial dysfunction [6,7]. Therefore, assessing the microvascular reactivity at the bedside using safe/validated devices becomes an attractive strategy for understanding the pathophysiology and therapeutics of sepsis.

The peripheral perfusion index (PPI) can be obtained from the photo-plethysmographic signal of an oximeter, which means two wavelengths of light (infrared/red) provide the display of a pulsatile waveform. PPI is the ratio of the pulsatile-to-non-pulsatile signal, expressed as a percentage. In healthy volunteers, the median PPI value was 1.4%, whereas lower values indicate low perfusion [8]. Therefore, changes in PPI reflect the vasomotor tone and are sensitive to detecting abnormal perfusion in critically ill patients [8].

Post-occlusive reactive hyperemia (PORH) refers to the increase in blood flow above baseline levels following a brief arterial occlusion [9]. It is a complex microvascular response to acute ischemia, and several factors have been proposed to explain it, such as metabolic vasodilators, endothelial vasodilators, myogenic response, and sensory nerves. PORH may represent a “reserve” or “recruitability” of vessel control ability by the vascular system when facing blood flow changes. However, since the precise mechanisms leading to PORH are unknown and lack a better definition, we called this ability to use a “reserve” of vascular control: *Peripheral Ischemic Microvascular Reserve* (PIMR).

Studies have shown that PORH is reduced in sepsis [6,7]. In a previous study, PORH was performed in septic shock, and PPI was used to estimate PIMR [10]. Interestingly, it was observed that patients with higher PIMR values died more when compared to patients with lower values characterizing an inverse relationship between PIMR and survival. This evidence suggests that the PIMR may be an independent risk factor for mortality. However, the original study [10] has some limitations, including being a monocentric study with a high mortality rate and lacking temporal evolution of changes in PPI. Thus, we decided to perform the present study. It was designed to test the validity of PIMR as a tool for prognostic enrichment at the bedside, as a new method for microcirculatory monitoring in sepsis, and to include clinical phenotypes other than septic shock.

## Materials and methods

### Study design, setting & participants

This observational study was conducted in four Brazilian ICUs between November/2020 and May/2022. All participants or their legal representatives provided written informed consent. The “Human Research Ethics Committee” of the Hospital de Clínicas, Federal University of Paraná, approved the research (protocol:3.913.982/2020). Furthermore, this study was conducted following the principles of the Declaration of Helsinki. This study is registered in the Brazilian Clinical Trials Registry as RBR-35tv9ft.

Consecutive patients (≥18 years) admitted to the ICU with sepsis diagnosis or within 24 hours after sepsis onset in patients admitted for other causes were enrolled. The exclusion criteria were pregnancy, severe hepatopathy (Child-Pugh class-C), severe coagulopathy (platelets < 20,000/mm3, the international normalized ratio > 2.0, or activated partial thromboplastin time > 70s), severe active bleeding, infective endocarditis, inaccessible perfusion assessment (severe hypothermia; Raynaud’s syndrome, peripheral arterial occlusive disease), and refusal to participate.

According to the most recent Sepsis consensus, this syndrome is defined as the presence of an infection associated with acute organ dysfunction (Sequential Organ Failure Assessment [SOFA] score ≥ 2 points) [11]. Septic shock remains a subgroup of sepsis wherein patients have hyperlactatemia (lactate ≥ 2 mmol/L), despite adequate fluid resuscitation and the requirement of vasopressors to maintain mean arterial pressure (MAP) ≥ 65 mmHg [11].

### Study protocol

All patients were treated following a standard protocol adapted from international guidelines [12]. Broad-spectrum antibiotics were administered within the first hour. The patients received fluid resuscitation with up to 30 mL/kg of crystalloids (first 3 hours of diagnosis). According to the physician’s criteria, if there was an individual clinical indication, the fluid resuscitation was maintained until there was a lack of reaction to the passive-leg test (the cutoff value to assess fluid responsiveness was an increase in cardiac output of 10%) or no respiratory variance of inferior vena cava diameter (the cutoff was 18% in mechanically ventilated patients and up to 42% in no mechanically ventilated patients) which were estimated using the Samsung Medison Ultrasound. If MAP remained < 65 mmHg, norepinephrine was used to normalize this hemodynamic parameter. Vasopressin was associated with refractory cases (noradrenaline dose > 0.5 μg/kg/h). Hemodynamic goals were established by these parameters: MAP ≥ 65 mmHg, diuresis > 0.5 ml/kg/h, and central venous oxygen saturation (ScvO_2_) > 70%.

The PIMR tests occurred after fluid resuscitation (within 24 h of diagnosis). The information collected included demographic, sepsis source, microbiology, chronic illnesses, Acute Physiology and Chronic Health Evaluation II (APACHE-II)/SOFA scores, hemodynamics, gasometric, and biomarkers. In addition, intensivists were blinded to PIMR values to avoid possible treatment bias. Finally, the patients were followed for 28 days after diagnosis or discharge.

The database and protocol of the present study are available in the Supporting information section (S1 Table and S1 File).

#### Assessment of capillary refill time (CRT)

CRT was defined as the time taken for the distal capillary bed to recover its color after moderate compression of the fingertip for 10 seconds, causing blanching. A delayed return of standard color ≥ 3 seconds indicated low perfusion [13,14].

#### Assessment of PIMR

**Fig 1:**
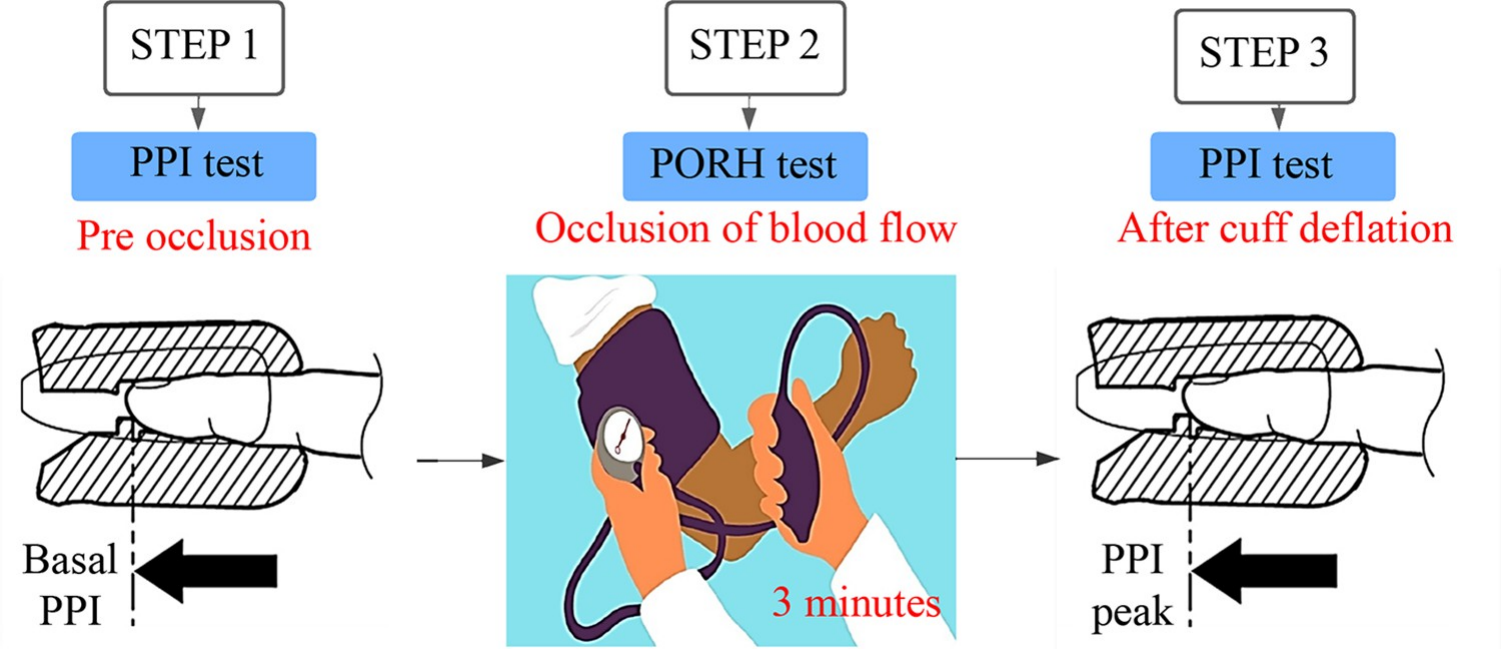
Assessment of PIMR assessed by the association between PPI/PORH methods. PIMR = peripheral ischemic microvascular reserve. PPI = Peripheral ischemic index. PORH = Post-occlusive reactive hyperemia.

It was assessed by the association between PPI/PORH methods (Fig 1). These tests were performed twice between 6–24 h and 24–48 h after sepsis diagnosis. First, PPI was measured by placing a probe (Masimo Radical, California-USA; MINDRAY, Shenzen-China) on the index finger. Then, after signal stabilization, PPI has recorded every 30 seconds for 5 minutes, and the average of the values was calculated (basal PPI). Subsequently, the PORH test was performed. First, a sphygmomanometer cuff was placed around the homolateral upper arm and was insufflated 50 mmHg above the systolic pressure to occlude the flow for 3 minutes. Next, after the cuff deflation, PPI values were recorded for 5 minutes, and the higher value was used (PPI peak).

Finally, the PIMR was determined using the ΔPPI peak (%), which was calculated using the following formula:

$$\Delta \mathrm{PPI}\;\mathrm{peak}=\frac{\mathrm{PPI}\;\mathrm{peak}‐\mathrm{PPI}\;\mathrm{basal}}{\mathrm{PPI}\;\mathrm{basal}}\times 100\%$$

After selection, patients were allocated to two groups: high PIMR group = ΔPPI peak ≥ 62%; low PIMR group = ΔPPI peak < 62%. This cutoff point was based on the ROC-curve analysis from a previous study [10]. Four trained researchers assessed the PIMR in the semi-recumbent position. The tests were performed in the upper limb without an intra-arterial catheter at 22 ˚C (ambient).

#### Outcomes

The primary outcome was the incidence of 28-day in-hospital mortality in the high and low PIMR groups in sepsis and its subgroups. The secondary outcomes included the prognosis of serial changes in ΔPPI peak (%) over the first 48 hours, the correlation between CRT (s) and ΔPPI peak values (%), and the multivariate analysis of PIMR as an independent predictor.

### Analytical approach

All data [15] were analyzed using the software IBM-SPSS Statistics 23 and GraphPad Prism-6. The Shapiro-Wilk test was used to verify the normality. Parametric data expressed as mean ± standard deviation (SD) was compared using Student’s t-test. The non-parametric data expressed as medians/interquartile ranges (IQR) were compared using the Mann-Whitney U-test. The categorical data were expressed as frequencies/percentages. Fisher’s test was performed to analyze the mortality rates. For the serial evaluation of the ΔPPI peak (%) over the first 48 hours, the association of the Mann-Whitney U test (intergroup analyses) and the Wilcoxon test (intragroup analyses) were performed. The Bonferroni post hoc test was used for multiple comparisons. A multivariate logistic regression model including variables with a p-value < 0.10 in univariate analyses and eliminating collinear variables was performed to analyze the high PIMR as an independent predictor. The goodness-of-fit of the final logistic regression model was assessed using the Hosmer–Lemeshow test. Finally, a correlation between continuous variables was performed using the Spearman test. All reported p-values were two-sided. A p-value < 0.05 was statistically significant.

The sample size was calculated based on previous local studies in sepsis [10,16]. Assuming 28-day in-hospital mortality of around 41%, we estimated a sample size of 226 patients to find a relative risk (RR) of 1.7 in the high-PIMR group with a sample power of 90%. The alpha error chosen was 0.05.

This study followed the STROBE guidelines.

## Results

**Fig 2:**
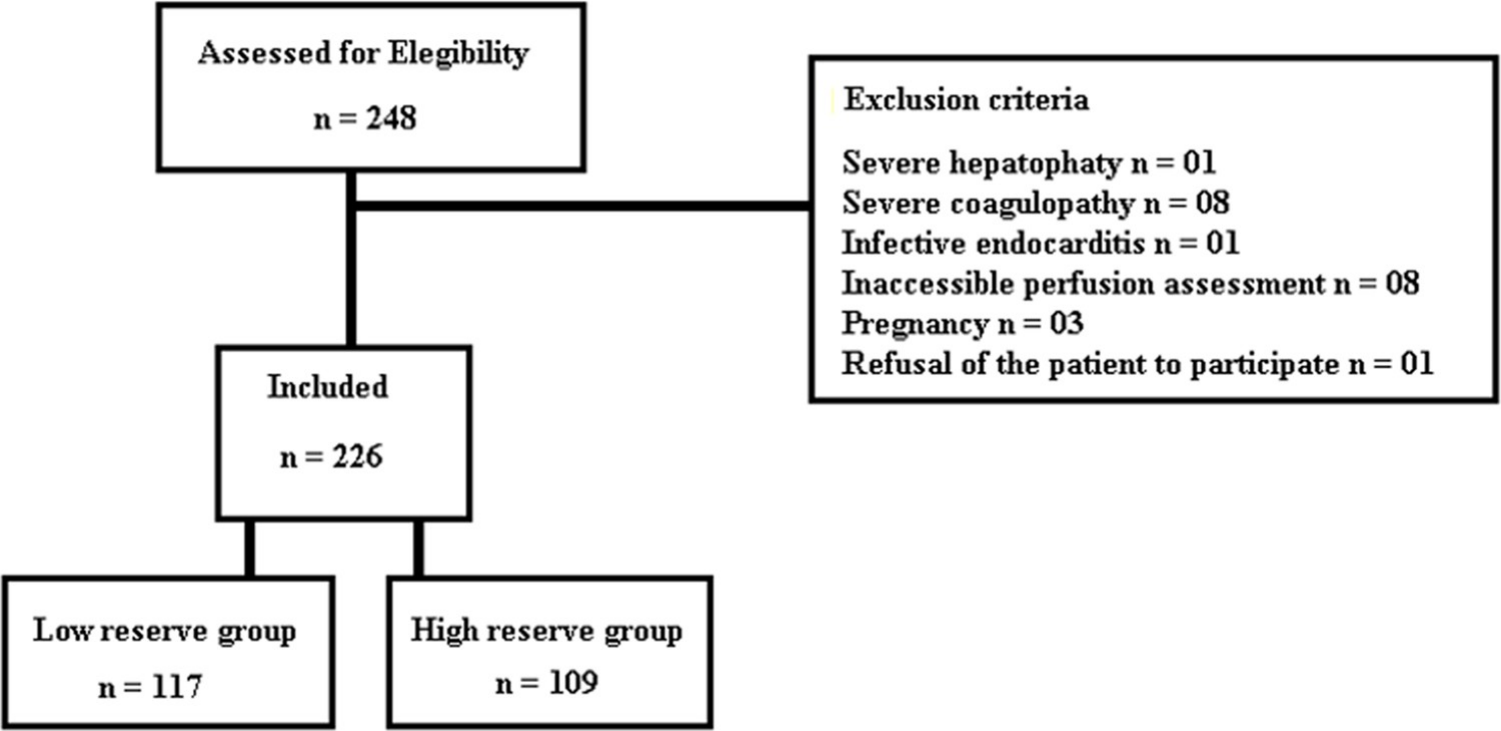
Flow chart of the study showing the group allocation based on the PIMR value (See Methods). PIMR = peripheral ischemic microvascular reserve.

Two hundred twenty-six patients with sepsis were included (Fig 2). The clinical, demographic, and hemodynamic data are presented in Table 1. No statistical differences were found between groups (high vs. low PIMR) in clinical data, severity scores (SOFA/APACHE II), blood biomarkers (procalcitonin, C-reactive protein, lactate), and vasoactive drugs. Moreover, both groups presented stable macro-hemodynamics. However, the high PIMR group had a higher incidence of patients with cerebrovascular disease, cancer, and abnormal peripheral perfusion (PPI and CRT) than the low PIMR group.

**Table 1:** Demographical and clinical characteristics of patients with after fluid resuscitation.

Variables	Whole Cohort N = 226	Low PIMR group N = 117	High PIMR group N = 109	p-value
**Clinical**				
Age, mean (SD), years	60 (16)	59 (16)	62 (15)	0.21
Sex, n (%)				0.10
Men	129 (57.1)	73 (62.4)	56 (51.4)	
Women	97 (42.9)	44 (37.6)	53 (48.6)	
Comorbidities, No. (%)				
Diabetes mellitus	72 (31.9)	38 (32.5)	34 (31.2)	0.88
Hypertension	126 (55.8)	66 (56.4)	60 (55)	0.89
Chronic kidney disease	30 (13.3)	16 (13.7)	14 (12.8)	1.00
Heart failure	39 (17.3)	19 (16.2)	20 (18.3)	0.72
Liver failure	10 (4.4)	5 (4.3)	5 (4.6)	1.00
Cerebral vascular disease	21 (9.3)	5 (4.3)	16 (14.7)	0.01*
Chronic pulmonary disease	36 (15.9)	23 (19.7)	13 (11.9)	0.14
Cancer	32 (14.2)	11 (9.4)	21 (19.3)	0.03*
Immunosuppression	45 (19.9)	18 (15.4)	27 (24.8)	0.09
**Source of infection, No. (%)**				
Respiratory	118 (52.2)	65 (55.6)	53 (48.6)	0.28
Abdominal	42 (18.6)	21 (17.9)	21 (19.3)	0.86
Urinary	27 (11.9)	12 (10.3)	15 (13.8)	0.32
Others	39 (17.3)	19 (16.2)	20 (18.3)	0.61
**Any microorganism in cultures,** No. (%)	156 (69)	85 (72.6)	71 (65)	0.25
**Confirmed bloodstream infection,** No. (%)	69 (30.7)	34 (29.3)	35 (32.1)	0.66
**Confirmed Covid 19 diagnosis,** No. (%)	34 (15)	22 (18.8)	12 (11)	0.13
**Scores and Biomarkers at ICU admission**				
SOFA score, mean (SD) ^a^	9 (4)	9 (4)	8 (4)	0.27
APACHE II score, mean (SD) ^b^	23 (8)	23 (9)	23 (8)	0.88
CRP, mean (SD), mg/dl	17.5 (11.4)	17.9 (11.7)	16.6 (8.5)	0.34
Procalcitonin, No./median (IQR), ng/ml	169/ 1.4 (0.4–6.8)	89 / 1.3 (0.3–6.6)	80 / 2 (0.5–7.1)	0.51
**Hemodynamic data after resuscitation**				
PAM, median (IQR), mmHg	83 (75–94)	83 (75–96)	85 (74–94)	0.84
Heart Rate, mean (SD), bpm	93 (22)	93 (23)	93 (21)	0.91
ScvO2, No./ mean (SD), %	75 / 74 (9)	40 / 75 (10)	35 / 73 (9)	0.39
Pv-aCo2, No./ median (IQR), mmHg	74 / 6.9 (2.9–9)	41 / 4.9 (2.3–8.7)	33 / 5.9 (3.4–10.3)	0.53
Arterial lactate, No./ median (IQR), mmol/L	220/ 1.9 (1.4–2.7)	114 /1.9 (1.4–2.5)	106/ 2 (1.5–3.2)	0.08
Urine Output, No./ median (IQR), ml/kg/h	210/ 0.5 (0.2–0.8)	108 / 0.5 (0.3–0.8)	102 / 0.5 (0.2–0.8)	0.96
**Vasoactive drugs use,** No (%)	116 (51.3)	63 (53.8)	53 (48.6)	0.50
**Norepinephrine dosage**, No./ median (IQR), μg/kg/min	114 / 0.2 (0.1–0.5)	62 / 0.2 (0.1–0.5)	52 / 0.3 (0.1–0.5)	0.09
**Vasopressin use,** No (%)	30 (13.3)	13 (11.1)	17 (15.,6)	0.33
**Abnormal peripheral perfusion**				
CRT (> 3 s), No (%)	82 (36.3)	26 (22.2)	56 (51.4)	< 0.01**
PPI < 1.4%, No (%)	84 (37.2)	14 (12)	70 (64.2)	< 0.01**
**Mortality,** No (%)	90 (39.8)	39 (33.3)	51 (46.8)	0.04*

*p < 0.05 **p < 0.01.

Abbreviations: APACHE, Acute Physiology, and Chronic Health Evaluation; CRP, C-reactive protein; CRT, Capillary refill time; IQR, interquartile Range; MAP, mean arterial pressure; PPI, peripheral perfusion index; Pv-aCO2, venous to arterial carbon dioxide difference; ***ScvO2***, central venous oxygen saturation; SD, standard variation; SOFA, Sequential Organ Failure Assessment.

Data are expressed as the mean (± standard deviation) for variables with normal distribution, median (interquartile range) for variables with skewed distribution, and number (percentage) for categorical variables.

Comparison between high and low-PIMR groups: Parametric data were compared using Student’s t-test; the non-parametric data were compared using the Mann-Whitney U-test, and the categorical data were compared using the Chi-square or Fisher’s tests.

^a^ Range, 0 to 24: Higher scores are associated with the intensity of organ dysfunction and higher mortality risk [11].

^b^ Range, 0 to 71: Higher scores are associated with the intensity of organ dysfunction and higher mortality risk [11].

The mortality was 40% (90/226), whose causes were refractory shock (45%), suspension of support/admission to palliative care (23%), multiple-organ dysfunction (21%), and worsening of underlying disease (11%). The comparison of the clinical demographic and hemodynamics between survivors and nonsurvivors is listed in the S2 Table. The nonsurvivors group was represented by a more significant portion of older patients with a history of immunosuppression, higher APACHE II and SOFA scores, higher procalcitonin levels, higher heart rate and arterial lactate levels, lower urine output, need higher norepinephrine doses and more often used vasopressin than survivors. In addition, in this group (nonsurvivors), abnormal perfusion (PPI < 1.4 and CRT > 3 s) and higher PIMR values were detected more frequently than in patients who did not die. There were no differences in other clinical data, infection source, confirmed culture, C-reactive protein, and other hemodynamic parameters between survivors and nonsurvivors.

**Fig 3:**
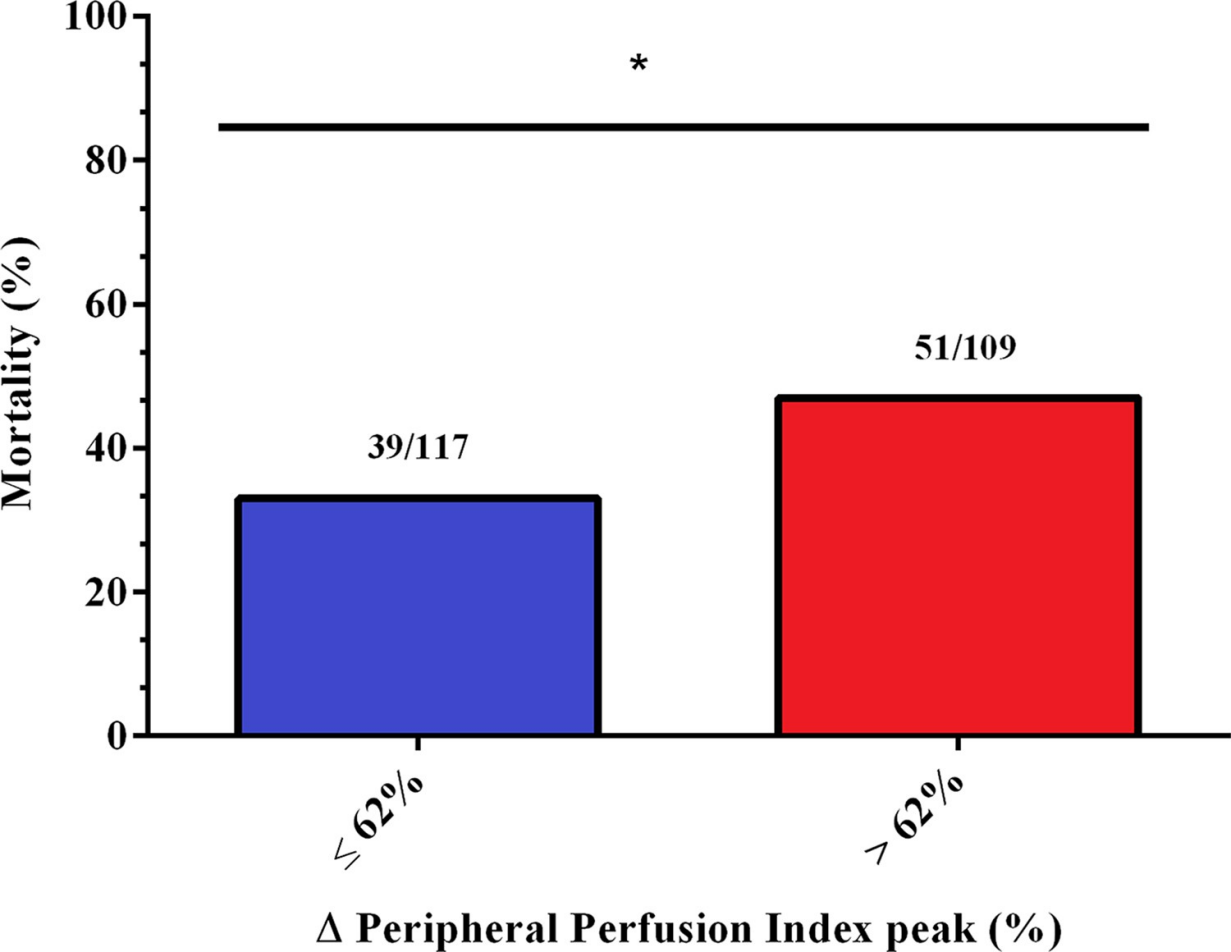
The 28-day in-hospital mortality rates in septic patients with high (> 62%) and low (< 62%) ΔPPI peak values. The numbers above bars are deaths/N of the respective group. * Significant difference between groups p = 0.04. High PIMR group N = 109. Low PIMR group N = 117. ΔPPI peak = variation of the peripheral perfusion index peak. PIMR = peripheral ischemic microvascular reserve.

As shown in Fig 3, we compared the mortality rates between high and low PIMR groups. Significant differences were observed between the groups (high vs. low PIMR), with higher mortality in the high PIMR group (RR 1.25; 95% CI, 1.00–1.55; p = 0.04).

As shown in multivariable analysis (Table 2), a high PIMR was an independent risk factor for predicting in-hospital mortality, with an Odds ratio around 2-fold higher than a low PIMR.

**Table 2:** Prognostic analysis of mortality in sepsis–Peripheral ischemic microvascular reserve.

	Bivariate					Multivariate
	Odds Ratio	95% CI	p-value	Odds Ratio	95% CI	p-value
**PIMR > 62%**	1.75	1.02–3.01	0.04*	2.15	1.13–4.11	0.02*
**Age**	1.02	1.00–1.04	0.01*	1.04	1.01–1.06	< 0.01**
**SOFA score**	1.21	1.12–1.31	< 0.01**	1.19	1.09–1.30	< 0.01**
**Heart rate**	1.03	1.01–1.04	< 0.01**	1.03	1.01–1.04	< 0.01**
**Lactate level**	1.50	1.20–1.89	< 0.01**	1.23	0.98–1.56	0.07

*p < 0.05 **p < 0.01.

Abbreviations: PIMR, Peripheral Ischemic Microvascular Reserve; SOFA: Sequential Organ Failure Assessment score.

**Fig 4:**
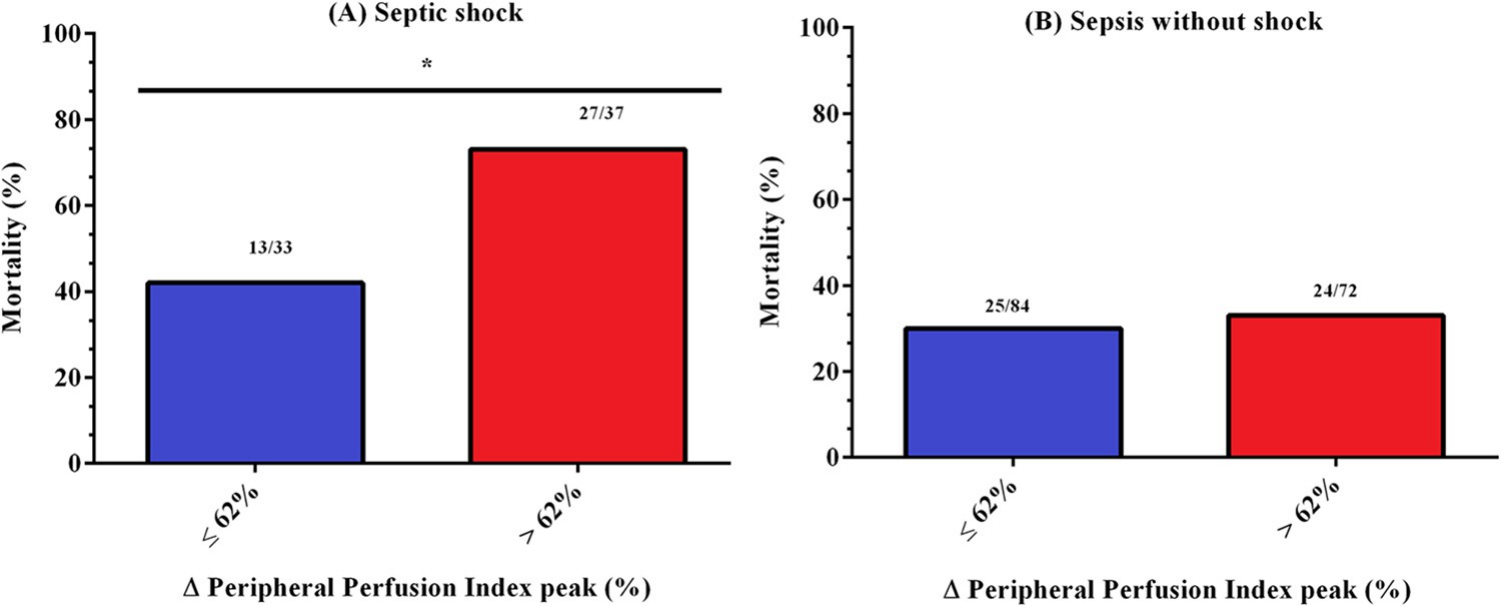
The 28-day in-hospital mortality rates in septic patients with high (> 62%) and low (< 62%) ΔPPI peak values in septic shock (Panel A) and sepsis without shock (Panel B). Septic shock patients N = 70. Sepsis without shock patients N = 156. The numbers above bars are deaths/N of the respective group. (A) * Significant difference between groups p = 0.01 using the Fisher’s test. (B) No significant difference between groups was observed for the sepsis without shock group p = 0.73. ΔPPI peak = variation of the peripheral perfusion index peak.

The prognostic value of PIMR also was analyzed in the sepsis subgroups. As illustrated in Fig 4A, the high PIMR group had higher mortality than the low PIMR group in septic shock (RR 2.14; 95% CI, 1.49–3.08, p = 0.01). However, as shown in Fig 4B, no difference was found between groups (p = 0.73) in sepsis without shock patients.

**Fig 5:**
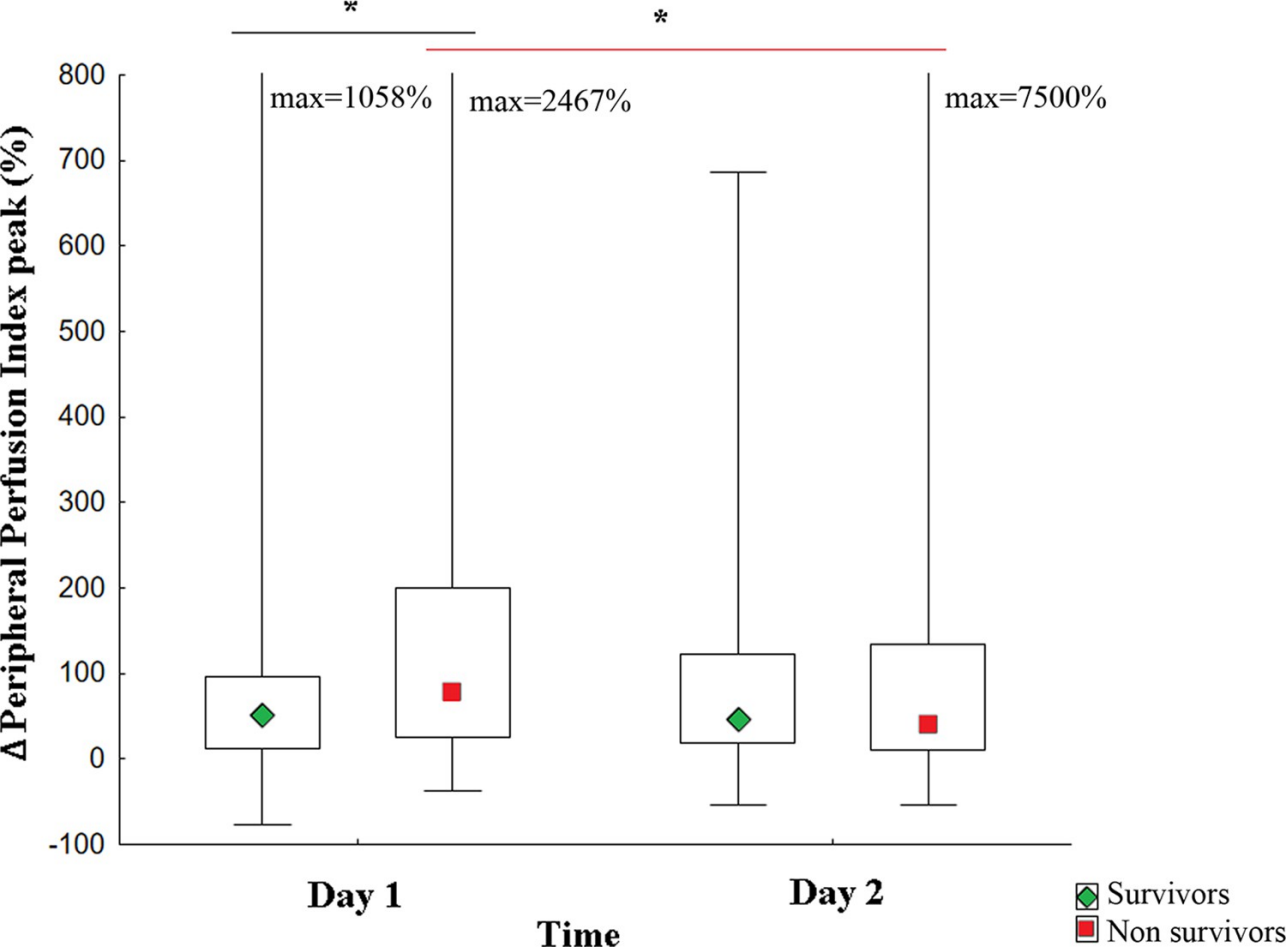
Temporal evaluation of the prognostic value of ΔPPI peak value in patients with sepsis. Patients with sepsis N = 226. Survivors in 28 days of follow-up N = 136. Nonsurvivors in 28 days of follow-up N = 90. Values are expressed as median/IQR. Intergroup analysis (survivors vs. nonsurvivors): Day 1* p = 0.01 and Day 2 p = 0.31. Intragroup analysis over 48 h: Survivors p = 0.52; Nonsurvivors * p = 0.02. **Statistical tests:** Mann-Whitney U test (intergroup analyses), the Wilcoxon test (intragroup analyses), and the Bonferroni post hoc test were used for multiple comparisons. ΔPPI peak = variation of the peripheral perfusion index peak.

As shown in Fig 5, we performed a serial assessment of ΔPPI peak values (%) over the first two days after resuscitation in patients with sepsis. There were significant differences in ΔPPI peak values between survivors and nonsurvivors within the first 24 hours of assessment (p = 0.01), whereby the nonsurvivors showed a higher median (ΔPPI peak = 78%, IQR 26–200%) than survivors (ΔPPI peak = 51.5%, IQR 12–95%). However, the difference was not observed on the second day of assessment (p = 0.31). In addition, these groups were analyzed separately, and we observed that the median significantly reduced (p = 0.02) over the 48 hours of observation for nonsurvivors (Day 1 = 78% IQR 26–200%, Day 2 = 40% IQR 11–135%). Nevertheless, there were no significant alterations in ΔPPI peak values over time for survivors (p = 0.52).

**Fig 6:**
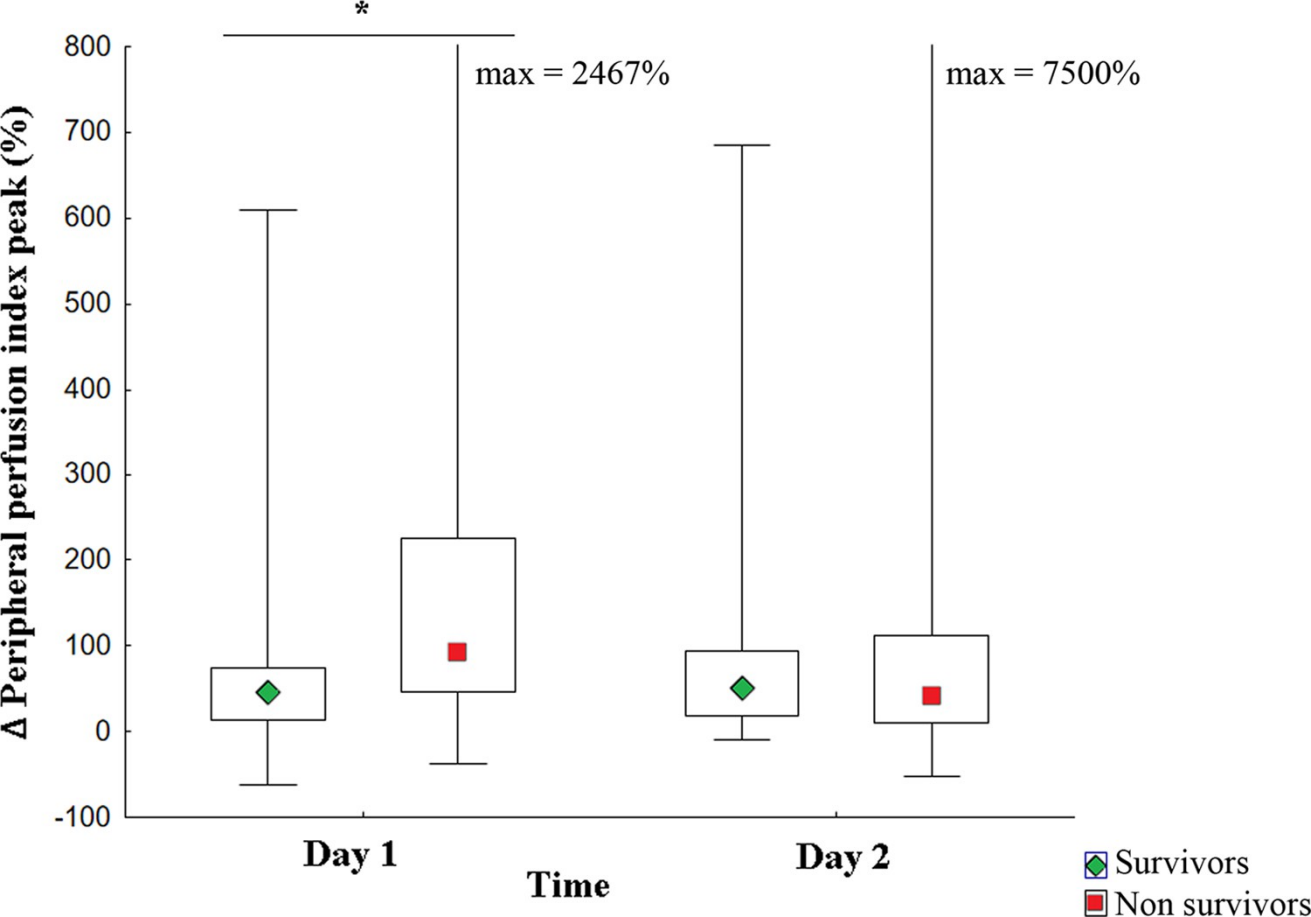
Temporal evaluation of the prognostic value of ΔPPI peak value in patients with septic shock. Septic shock patients N = 70. Survivors in 28 days of follow-up N = 29. Nonsurvivors in 28 days of follow-up N = 41. Intergroup analysis (survivors vs. nonsurvivors): Day 1* p = 0.03 and Day 2 p = 0.56. Intragroup analysis over 48 h: Survivors p = 0.72; Nonsurvivors p = 0.12. **Statistical tests:** Mann-Whitney U test (intergroup analyses), the Wilcoxon test (intragroup analyses), and the Bonferroni post hoc test were used for multiple comparisons. ΔPPI peak = variation of the peripheral perfusion index peak.

Additionally, the same serial analysis was performed over the first 48 hours of the follow-up in septic shock patients (Fig 6). Similar to the entire group of patients with sepsis, intergroup analysis (survivors vs. nonsurvivors) showed significant differences in ΔPPI peak (%) values within the first 24 hours (p = 0.03), which were not observed on the second day of evaluation (p = 0.56). Moreover, ΔPPI values were not significantly altered for both groups over time (survivors p = 0.72; nonsurvivors p = 0.12).

**Fig 7:**
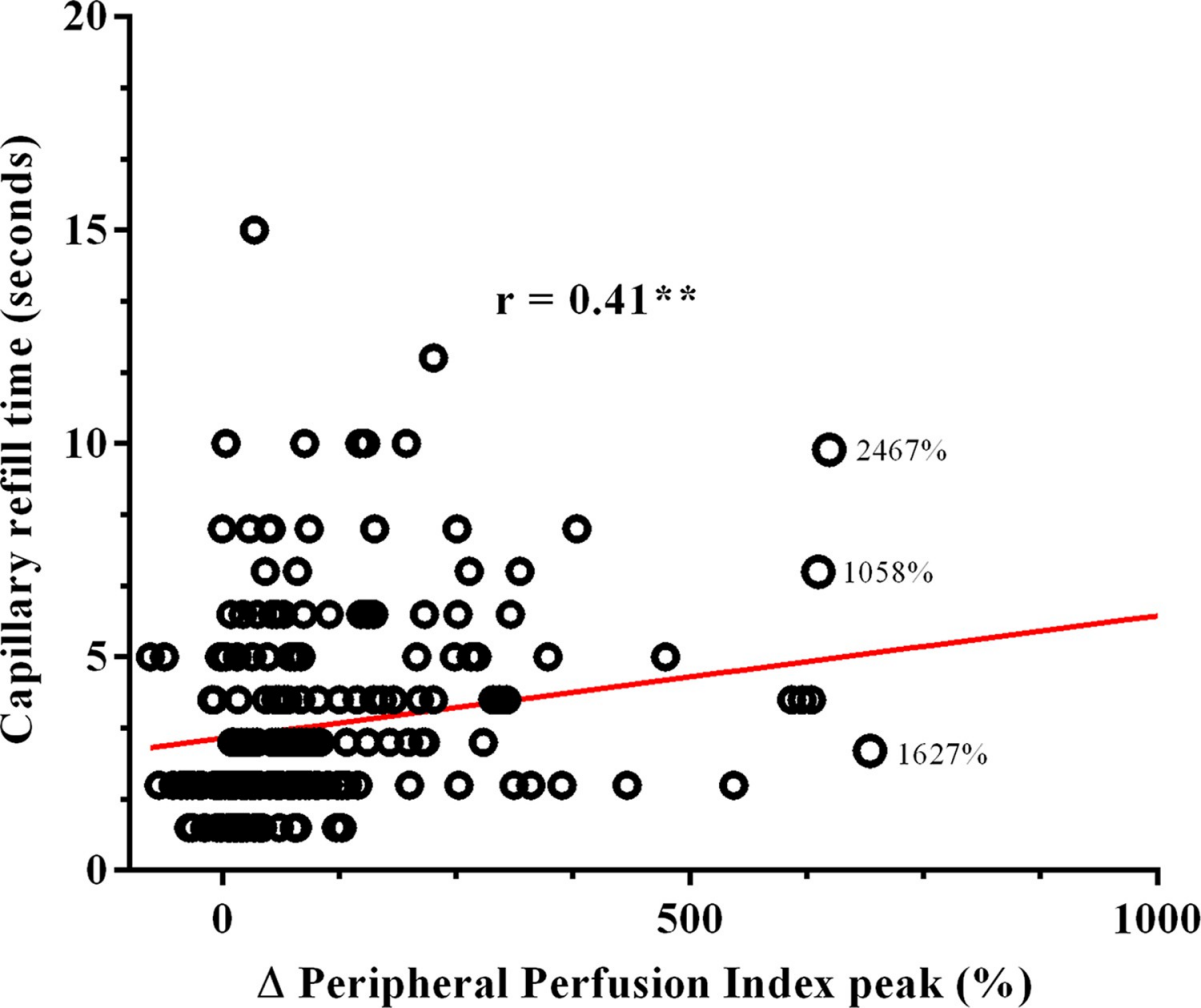
Correlation between ΔPPI peak (%) and CRT (s) values within 24 h of sepsis diagnosis. **p < 0.01. **Statistical test:** Spearman test. Patients with sepsis N = 226. ΔPPI peak = variation of the peripheral perfusion index peak. CRT = Capillary refill time.

Fig 7 shows a moderate positive correlation (r = 0.41) between ΔPPI peak (%) and CRT values within the first 24 hours of diagnosis (p < 0.01).

## Discussion

The main results of the present reports are: i) there is a relationship between PIMR and mortality in sepsis, mainly in the septic shock subgroup; ii) this prognostic enrichment is valid if obtained up to 24 hours after resuscitation; iii) this prognostic enrichment can be easily obtained at the bedside and iv) PIMR seems to be an easy test for the early detection of at least two subgroups with different mortality risks and the development of future guided-therapy.

Previous results from our group suggested that PIMR could discriminate between survivors and nonsurvivors even when several other variables were identical between the patients [10]. The present results confirmed the former suggestion and expanded the prognostic enrichment of PIMR, mainly when obtained earlier (within 24 hours) after resuscitation.

Among the microcirculatory disorders in sepsis, several studies have demonstrated the relevance of peripheral hypoperfusion to mortality [5,17]. However, the role of vascular hyporesponsiveness in the resulting hypoperfusion is less known. Studies performed in the skeletal muscle [6] and fingertips [7] have shown a possible causality related to the known functional endothelial/vascular damage induced by sepsis [18].

The skin and its abundant microcirculation make it readily amenable to noninvasive bedside measures. The PORH is a well-established test proposed to evaluate microvascular reactivity/endothelial function [19]. However, some essential questions must be considered. First, PORH magnitude is an apparently organ-dependent phenomenon [6,7,20] and, thus, depends on different metabolic pathways in different tissues. Second, the test interpretation also depends on the phase of the hyperemic response evaluated because early flow responses seem to be derived mainly from mechanosensitive mechanisms, while shear stress and metabolic factors affect late flow response [21].

Several studies [6,7] pointed out that septic patients have a lower PORH response attributed to endothelial (and probably vascular) dysfunction in sepsis [18,22]. Therefore, it was somewhat surprising that our results showed that patients with a more significant vasodilatation response after limb ischemia exhibited higher mortality. Although we do not have a definite explanation for this difference, some mechanisms may be operative. One possibility refers to the mechanical aspect of the test. As mentioned before, PIMR may reflect the proportion of recruitable capillaries, arterioles, and meta-arterioles after the transitory ischemic stimulus [6]. Thus, the more severely ill patients, especially those with shock, may have unused microvascular vasodilatory reserve despite flow-dependent hypoxia. Conversely, patients with a better prognosis may be more efficient in coupling blood flow to the tissue demands, thus evoking less microvascular reserve when facing hypoxia. The present study verified a moderate positive correlation between PIMR and CRT values, indicating that the higher the peripheral perfusion (low CRT), the lower the measured PIMR.

A second possibility to explain the inverse correlation between PIMR and mortality would be a metabolic/inflammatory component. Although the pathophysiological mechanisms of PORH in the skin are not fully understood, a report showed that the expression and release of neuropeptides via sensory nerves and derivatives from cytochrome P-450 epoxygenases seem to play an essential role in the peak/timing of hyperemia [23,24]. Additionally, the neuropeptide substance P and calcitonin gene-related peptide are known markers of severity in sepsis [25–27], although we recently demonstrated no significant correlations between these neuropeptides and PIMR in septic patients [28]. One also must remember that sepsis exhibits an essential inflammatory response [29]. Nitric oxide and other inflammatory mediators, such as reactive oxygen species and local cytokines, may be operatives. Patients with a worse prognosis may have more putative mediators in the microcirculatory beds, thus showing more significant vasodilation following reperfusion. Since the systemic blood pressure is low, the host will mobilize endogenous vasoconstrictor mechanisms such as the adrenergic system [30]. It has been shown that the more robust activation of the adrenergic system in sepsis may lead to desensitization of adrenergic receptors, thus contributing to hypotension and adrenergic hyporesponsiveness [31]. If this holds in the clinical setting, a higher PIMR should indicate a failure to counteract the hypoxia-induced vasodilation in nonsurvivors. On the other hand, we demonstrated that increasing the dose of norepinephrine in patients with shock increases the PIMR [10]. Thus, new studies deserve to be conducted to evaluate the relationship between the adrenergic system and the PIMR.

Sepsis is a heterogeneous syndrome induced by several microorganisms and affects hosts with specific genetics/comorbidities whose time-to-diagnosis and treatment differ. This fact results in distinct therapeutic responses [2]. Therefore, to gain further insight into the potential use of PIMR in a precision medicine approach, we verified that the high PIMR group showed two times higher mortality than the low PIMR group in the septic shock subgroup. In contrast, such differences were not observed in patients bearing sepsis without shock, suggesting that septic shock was an effective mediator. However, further formal analyses need to estimate this mediation magnitude accurately.

Sepsis is also known to cause changes in macro and microvascular reactivity related to dynamic and time-dependent pathways [32]. Therefore, we evaluated PIMR at two different time points (24 and 48 hours). Our results showed that the PIMR differentiated survivors from nonsurvivors only in the first 24 hours, losing its predictive capacity on the second day after shock onset. Interestingly, the period of the most significant predictive value of PIMR for mortality in shock coincides with the period in which the most severely ill patients have a higher cardiac index and lower vascular resistance [33]. Further studies comparing these parameters should be performed to confirm this interaction between macro and micro-hemodynamics.

Early identification of potential nonsurvivors for personalized management remains an attractive idea in sepsis. Furthermore, our results provided new perspectives for diagnosing microvascular disturbances and future-guided management. In this sense, the PIMR values could point to the subgroup where fluid resuscitation and vasoactive drugs should be used with more outstanding care, thus avoiding fluid overload and catecholamine excess, known clinical worsening factors, in those lower-risk patients [34,35]. Lastly, the relationship between PIMR and adrenergic stimulation could contribute to monitoring sympathetic modulation, a treatment of recent interest in human sepsis [36].

Our study has limitations. First, due to the Covid-19 pandemic, the sample was recalculated because it verified a change in pre-study predicted mortality (estimated 47%/ final 41%). Considering that the speed of study entry differed among the centers, the final sample did not have the same proportion of patients. Second, the proposed design was non-interventional, so the association between high PIMR and mortality does not prove causality. Third, it cannot be said that the same tissue response in non-vital organs will occur in vital organs. Comparative measurement is needed, although previous studies suggest this may occur [37]. Finally, the uncertain timing of sepsis onset before ICU admission limits the extrapolation of our findings to other settings outside ICUs.

In conclusion, although the advances in imaging techniques allow direct microcirculation visualization [38], the high costs associated with specific training limit the dissemination of its clinical practice. Thus, even though we do not have definite mechanisms to explain it, PIMR assessment using the PPI/PORH combination has advantages, including fast bedside assessment which can be performed several times, low cost, and, more importantly, good predictive quality for survival and it represents a potential prognostic enrichment tool to be adopted in clinical routine.

## Supporting information

S1 Table Database. All Patients.
(SAV)

S2 Table Demographical and clinical characteristics of patients with sepsis after fluid resuscitation. (PDF)

S1 File Study protocol. (PDF)
