# Supplementary material for: Peripheral ischemic reserve in sepsis and septic shock as a new bedside prognostic enrichment tool: A Brazilian cohort study
Source: PLoS One. 2023 Jul 5;18(7):e0288249. doi: 10.1371/journal.pone.0288249 (PMC10321605; doi:10.1371/journal.pone.0288249)
Supplement: S2 Table — (PDF) [file pone.0288249.s002.pdf]

S3 Table. Demographical and Clinical Characteristics of patients with sepsis after fluid resuscitation

**Variables**	**All patients n = 226**	**Survivors n = 136**	**Non survivors n = 90**	**P-value**
**Clinical**				
Age, mean (SD), years	60 (16)	58 (16)	63 (15)	0.01*
Sex, n (%)				1.00
Men	129 (57.1)	78 (57.4)	51 (56.7)	
Women	97 (42.9)	58 (42.6)	39 (43.3)	
Comorbidities, No. (%)				
Diabetes mellitus	72 (31.9)	42 (30.9)	30 (33.3)	0.77
Hypertension	126 (55.8)	72 (52.9)	54 (60)	0.34
Chronic kidney disease	30 (13.3)	15 (11)	15 (16.7)	0.23
Heart failure	39 (17.3)	20 (14.7)	19 (21.1)	0.21
Liver failure	10 (4.4)	4 (2.9)	6 (6.7)	0.20
Cerebral vascular disease	21 (9.3)	11 (8.1)	10 (11.1)	0.49
Chronic pulmonary disease	36 (15.9)	19 (14)	17 (18.9)	0.36
Cancer	32 (14.2)	17 (12.5)	15 (16.7)	0.44
Immunosuppression	45 (19.9)	20 (14.7)	25 (27.8)	0.02*
**Source of infection, No. (%)**				
Respiratory	118 (52.2)	65 (47.8)	53 (58.9)	0.08
Abdominal	42 (18.6)	26 (19.1)	16 (17.8)	0.86
Urinary	27 (11.9)	20 (14.7)	7 (7.8)	0.10
Others	39 (17.3)	25 (18.4)	14 (15.6)	0.52
**Any microorganism in cultures,** No. (%)	156 (69)	93 (68.4)	63 (70)	0.88
**Confirmed bloodstream infection,** No. (%)	69 (30.7)	41 (30.1)	28 (31.5)	0.88
**Scores and Biomarkers at ICU admission**				
SOFA score, mean (SD) [a]	9 (4)	7 (4)	10 (4)	< 0.01**
APACHE II score, mean (SD) [b]	23 (8)	21 (7)	27 (8)	< 0.01**
CRP, mean (SD), mg/dl	17.5 (11.4)	17 (12)	18.6 (9.9)	0.25
Procalcitonin, No./median (IQR), ng/ml	169/ 1.4 (0.4-6.8)	93 / 1.1 (0.3-3.8)	76 / 3.5 (0.7-10.5)	< 0.01**
**Hemodynamic data after resuscitation**				
PAM, median (IQR), mmHg	83 (75-94)	83 (74-97)	84 (75-92)	0.87
Heart Rate, mean (SD), bpm	93 (22)	85 (16)	101 (21)	< 0.01**
ScvO2, No./ mean (SD), %	75 / 74 (9)	37 / 75 (9)	38 / 73 (10)	0.36
Pv-aCo2, No./ median (IQR), mmHg	74 / 6.9 (2.9-9)	35 / 4.9 (3.9-9)	39 / 5.2 (2.4-10.9)	0.82
Arterial lactate, No./ median (IQR), mmol/L	220/ 1.9 (1.4-2.7)	132 / 1.7 (1.3-2.5)	88 / 2.3 (1.7-3.5)	< 0.01**
Urine Output, No./ median (IQR), ml/kg/h	210/ 0.5 (0.2-0.8)	123 / 0.6 (0.4-0.9)	87 / 0.4 (0.1-0.7	< 0.01**
**Vasoactive drugs use,** No (%)	116 (51.3)	53 (39)	63 (70)	< 0.01**
**Norepinephrine dosage**, No./ median (IQR), µg/kg/min	114 / 0.2 (0.1-0.5)	52 / 0.2 (0.1-0.4)	63 / 0.3 (0.1-0.6)	< 0.01**
**Vasopressin use,** No (%)	30 (13.3)	10 (7.4)	20 (22.2)	< 0.01**
**Abnormal peripheral perfusion**				
CRT (> 3 s), No (%)	82 (36.3)	41 (30.1)	41 (45.6)	0.02*
PPI < 1.4%, No (%)	84 (37.2)	35 (25.7)	49 (54.4)	< 0.01**
**PIMR,** median (IQR), **%**	60 (17-133)	51 (12-97)	78 (25-202)	< 0.01**

*p < 0.05 **p < 0.01.

Abbreviations: APACHE, Acute Physiology and Chronic Health Evaluation; CRP, C-reactive protein; CRT, Capillary refill time; IQR, interquartile range; MAP, mean arterial pressure; PPI, peripheral perfusion index; Pv-aCO2, venous to arterial carbon dioxide difference; ScvO2, central venous oxygen saturation; SD, standard variation; SOFA, Sequential Organ Failure Assessment.

Data are expressed as the mean (± standard deviation) for variables with normal distribution, median (interquartile range) for variables with skewed distribution, and number (percentage) for categorical variables.

Comparison between high and low-PIMR groups: parametric data were compared using Student's t-test; the non-parametric data were compared using the Mann-Whitney U-test, and the categorical data were compared using the Chi-square or Fisher's tests.

[a] Range, 0 to 24: higher scores are associated with the intensity of organ dysfunction and higher mortality risk (11).

[b] Range, 0 to 71: higher scores are associated with the intensity of organ dysfunction and higher mortality risk (11).
