# Supplementary material for: Peripheral ischemic reserve in sepsis and septic shock as a new bedside prognostic enrichment tool: A Brazilian cohort study
Source: PLoS One. 2023 Jul 5;18(7):e0288249. doi: 10.1371/journal.pone.0288249 (PMC10321605; doi:10.1371/journal.pone.0288249)
Supplement: S1 File — (PDF) [file pone.0288249.s003.pdf]

STUDY PROTOCOL

EVALUATION OF THE PERIPHERAL MICROVASCULAR RESERVE IN SEPSIS

(DAY 01 --- FIRST 24 HOURS)

PATIENT CODE: __________
DATA:_______________________________

BASELINE DATA
FC BASAL: _________ PAM: PVC: ________ Lactate arterial: _____________
Medications in use:
____
Mechanical ventilation parameters:
____
Time (seconds):

0	30	60	90	120	150	180	210	240	270	300

PRE-Occlusion VASCULAR STATUS
a) CAPILLARY FILLING 5 CHIRODACTILOs: ________
(b) PULSE RADIAL_______________________________
c) ULNAR PULSE ______

3-MINUTE ARTERIAL Occlusion *

IP AFTER DEFLATION
** MAXIMUM IP (PEAK IPP): ________

Time (seconds):

0	30	60	90	120	150	180	210	240	270	300

POST-Occlusion VASCULAR STATUS

a) CAPILLARY FILLING 5 QUIRODACTILOs: ___________
b) RADIAL PULSE ____

c) ULNAR PULSE ______
d) FC AFTER: ______
e) IP MISSING IN SOME FINGER APOS TEST? ______________________

EVALUATION OF THE PERIPHERAL MICROVASCULAR RESERVE IN SEPSIS

(DAY 02 --- BETWEEN 24 AND 48 HOURS)

PATIENT CODE:_____
DATA:____________________________

BASELINE DATA
FC BASAL: _________ PAM: PVC: ________ Lactate arterial: _____________
Medications in use:
____

Mechanical ventilation parameters:
____

Time (seconds):

0	30	60	90	120	150	180	210	240	270	300

PRE-Occlusion VASCULAR STATUS

a) CAPILLARY FILLING 5 CHIRODACTILOs: ________
(b) PULSE RADIAL_______________________________
c) ULNAR PULSE ______

3-MINUTE ARTERIAL Occlusion ***

IP AFTER DEFLATION

** MAXIMUM IP (PEAK IPP): ________

Time (seconds):

0	30	60	90	120	150	180	210	240	270	300

POST-Occlusion VASCULAR STATUS

a) CAPILLARY FILLING 5 QUIRODACTILOs: ___________
b) RADIAL PULSE ____
c) ULNAR PULSE ______

d) FC AFTER: ______
e) IP MISSING ON SOME FINGER AFTER TEST? ________________________

EVALUATION OF THE PERIPHERAL MICROVASCULAR RESERVE IN SEPSIS

Patient code: ________
Data: ____________________
Name:____

Age: _________

Registration No HC:____
Sugisawa Hospital Registry: ____

ICU admission:_____

Reason for hospitalization in CHC-UFPR:
____
____

Comorbidities
____
____

Start date of sepsis _______________Origem sepsis: ________
Etiology sepse____________________________
Estimated weight (kg) __
Pcr (mg/dl) _________________________
Procalcitonina (mcg/l) _______

Apache II score:

Data Patient admission/or day of diagnosis of sepsis	
Temperature	
PAM	
FC	
FRI	
PaO2/Fio2	
Ph/HCO3	
On	
K	
Cr	
VG	

Leukocytes	
Glasgow	
Age	
Chronic problems (**1) Cirrhosis 2) Niha Class IV 3) severe COPD or pulmonary hypertension 4) On regular dialysis or 5) Immunosuppressed)**	
Total	

SOFA Score

D1 (First 24 hours of diagnosis of sepsis)		D2 (24-48 hours post diagnosis of sepsis)	
Po2/Fio2		Po2/Fio2	
Platelets		Platelets	
PAM/TWO		PAM/TWO	
Bilirubin		Bilirubin	
GLASGOW		GLASGOW	
CREATININE/DU		CREATININE/DU	
TOTAL		TOTAL	

STATUS AFTER HEMODYNAMIC RESUSCITATION (FIRST 24 HOURS)

VASOACTIVE DRUGS AFTER HEMODYNAMIC RESUSCITATION D1 (mmol/L) (QUAL E DOSE)
____

CENTRAL VENOUS SATURATION The$_2$ (%) POST HEMODYNAMIC RESuscitaTION D1 _____________

FIRST URINE OUTPUT 24 HOURS (ml/kg/h)
____

ARTERIAL SERUM LACTATE AFTER HEMODYNAMIC RESuscitaTION D1 (mmol/L) ________

ARTERIAL Serum LACTATE D2 (mmol/L) __________

GAP CO2 (mmHg) POST HEMODYNAMIC RESUSCITATION D1
____
